# Spatial Biology Evolution: Past, Present and Future of Mapping Life in Context

**DOI:** 10.3390/cells15090743

**Published:** 2026-04-22

**Authors:** Alexander E. Kalyuzhny

**Affiliations:** Dental Basic Sciences, Division of UMN Twin Cities, University of Minnesota, 17-252 Moos Tower 515 Delaware St SE, Minneapolis, MN 55455-0357, USA; kalyu001@umn.edu

**Keywords:** spatial biology history, spatial omics, multiplexed immunofluorescence (mIF), spatial transcriptomics (ST), spatial proteomics (SP), tumor microenvironment (TME), cyclic immunofluorescence (CyCIF), sequential immunofluorescence (seqIF), fast fluidic exchange (FFeX), histochemistry, IHC

## Abstract

**Highlights:**

**What are the main findings?**

- Methodological Lineage and Evolution. The perspective traces the spatial revolution from its 19th-century botanical foundations (François-Vincent Raspail) to the 1941 inception of antibody-based detection (Albert Hewett Coons), culminating in modern automated platforms that achieve subcellular resolution for 40+ markers.
- Technological Convergence: A critical analysis of the current “spatial trilemma”—the trade-off between spatial resolution, analytical throughput, and transcriptomic depth—highlights how new platforms like Akoya, Visium HD and COMET™ are narrowing these gaps.

**What are the implications of the main finding?**

- Standardization of Multi-Omic Workflows. The perspective establishes a framework for synchronizing disparate data layers (mRNA, protein, and metabolites) on a single tissue section, which is essential for decoding the “neighborhood effects” that dictate cell function and disease progression.
- Translational Impact on Precision Medicine. By moving from tissue homogenization to molecular cartography, these findings provide a roadmap for drug discovery and clinical diagnostics, specifically in identifying physically or chemically shielded tumors and mapping drug–target interactions in their native environments.

**Abstract:**

The life sciences are currently undergoing a serious transition from the reductive biochemical analysis of dissociated tissues to non-destructive “spatial forensics”. In addition to discovering new molecules, we are moving towards finding out their precise tissue localization and performing in situ interrogation to uncover a biological logic within preserved cellular “neighborhoods”. Our perspective is focused on exploring the spatial imperative, including the structural logic and “neighborhood effects” of the tissue microenvironment, which is a prerequisite to understanding cellular function in normal and in pathological conditions. Beginning with a historical foundation of the origins of histochemistry, dating back to the 19th century with pioneer botanist François-Vincent Raspail, we emphasize the technological metamorphosis, transitioning from classical immunohistochemistry to modern multi- and high-plex spatial multi-omics. A critical evaluation of the current operational landscape has been made, addressing the engineering strategies behind multiplexed immunofluorescence (mIF), the challenges of experimental design in spatial transcriptomics, and the functional symbiosis between targeted and unbiased spatial proteomics. There are many layers of genomic and proteomic information we have to consider in order to unravel the mechanisms underlying body function. If we learn how to combine all this information together, we will be able to better understand how cells communicate with each other and what disrupts their communication, leading to cancer and many other pathologies. It is obvious that by implementing spatial biology tools, it becomes possible to develop new medicines and treat diseases in the most efficient ways. At the same time, we realize that there is an urgent need to learn how to put data pieces together so that they blend seamlessly into a meaningful output, further transitioning spatial biology over time into a routine tool to cure for both common and rare diseases and improve our lives and health.

## 1. Introduction

Understanding the complex nature and function of living organisms requires a systemic approach to analyze the interactions between molecular players in context [1]. A breakthrough in developing context-analyzing platforms led to the introduction of spatial biology, a new discipline transforming the study of biological systems by moving beyond traditional single-cell analyses. In general, spatial biology is a method of collecting data from multiple tissue targets to create a comprehensive overview of complex biological organization. Being non-destructive in nature, spatial biology can anchor molecular interactions within their functional architecture. By bypassing the limitations of tissue homogenization, which erases the “neighborhood” effects of the microenvironment, spatial biology preserves the structural logic of the tissue. Spatial biology is changing the way we study living things by looking at tissues as a whole system rather than just a mix of different parts. Instead of merely examining gene expression or protein localization, we are now attempting to comprehend the synergistic interactions of these components within their natural environment. This is important because the location of a cell often determines its function, and the spatial arrangement of molecules ultimately controls development and disease progression. By studying these dynamics in their natural contexts, we can gain a deeper understanding of how the spatial arrangement of molecules affects the way our bodies work when we are healthy and when we are ill. This approach helps us move beyond merely observing things and instead gives us a better understanding of how spatial relationships impact our overall health. This perspective seeks to move from the foundational history of spatial biology towards a critical evaluation of its current trajectory and untapped potential. Rather than retracing the well-trodden paths of the existing literature, our analysis prioritizes the “friction points” of the field: the complex synchronization of multi-omics data layers and the persistent hurdles in cross-platform standardization. By focusing on these logistical realities, we aim to clarify how spatial insights are being converted into tangible assets for drug discovery and the broader landscape of precision medicine. This article is not just a simple summary; it provides a detailed look at the spatial revolution from different angles. It is meant for experts who wish to dive into tough issues, such as making sense of different types of data, while also providing a clear overview to those who are not specialists. By connecting basic ideas to more complex problems, it is hoped that people will understand how spatial context is changing the life sciences. It is like building a bridge between the basics and advanced concepts so that everyone can see how spatial context is redefining the field. This can help spark a wider understanding of what is going on and how it is impacting the life sciences.

## 2. Foundations of Spatial Analysis: From Microscopy to Multiplexing

### 2.1. Historic Overview

Spatial biology has a rich history that began with François-Vincent Raspail, a French botanist, physician, and politician. He is often called the “Father of Histochemistry” because of his groundbreaking work. Raspail believed that chemistry should be studied in the context of living cells, which laid the foundation of modern spatial biology. In 1830, he published a paper titled “Essai de chimie microscopique appliquée à la physiologie,” in which he explained his ideas on histochemistry [2]. He was a pioneer in using the microscope as a tool to study the chemical makeup of cells, not just to observe them. Raspail proposed that cells are the basic building blocks of life and that all growth occurs through cell development. This was a big idea, and it came before other scientists, such as Schwann and Schleiden, had similar thoughts. He also anticipated the famous phrase “Omnis cellula e cellula,” which means “all cells come from cells.” This phrase was later coined by Rudolf Ludwig Carl Virchow, a German biologist known as the “father of modern pathology.” Raspail was the first to use chemical reagents to analyze the molecular components of plant tissue sections. One of his most famous experiments used the starch–iodine reaction to detect starch granules in plant cells. He also developed tests to detect proteins and lipids and showed that these substances are distributed in complex patterns within tissues. Raspail’s work was important and paved the way for our understanding of spatial biology. He can be considered a pioneer in cell theory, and his ideas about the importance of cells in growth and development were well ahead of their time. Raspail’s use of chemical reagents to study plant tissue sections was a major breakthrough that allowed him to gain a deeper understanding of the molecular components of cells. His work on the detection of starch granules, proteins, and lipids was also significant, showing that these substances are not distributed evenly within tissues. Instead, they are found in complex patterns specific to each tissue type. Raspail’s legacy continues to influence the field of spatial biology, and his ideas about the importance of cells and their chemical makeup remain widely accepted today. His use of the microscope as a tool to study the chemical properties of cells was a major innovation that paved the way for many later discoveries in the field. Overall, Raspail’s work was important and laid the foundation for our understanding of spatial biology today. It is worth noting that Raspail’s work was not limited to just one area of study. He was a true pioneer, and his ideas and discoveries had a major impact on several fields. He is still remembered today as one of the most important scientists of his time, and his legacy continues to inspire new generations of researchers. In conclusion, François-Vincent Raspail was a pioneer in spatial biology. His work on the importance of cells and their chemical makeup laid the foundation for many later discoveries, and his use of the microscope as a tool to study the chemical properties of cells was a significant innovation. His legacy continues to influence the field of spatial biology, and his ideas and discoveries remain widely acknowledged.

### 2.2. The Legacy of Immunohistochemistry and Immunofluorescence

**Figure 1:**
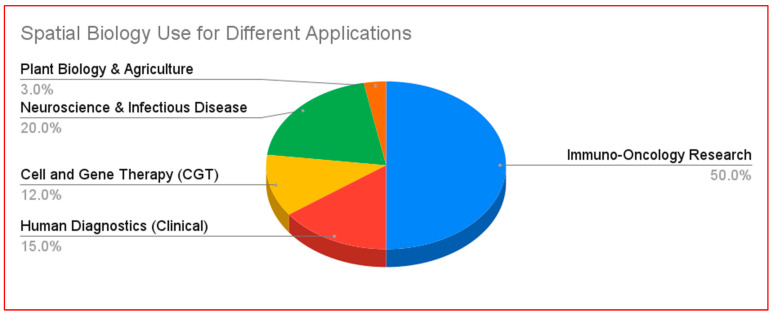
Estimated frequency of spatial biology use for different biological and medical applications.

The trajectory toward modern spatial biology gained decisive momentum eight decades ago, as classical histochemistry provided the essential scaffolding for what eventually matured into immunohistochemistry. Such a metamorphosis of general histochemistry into targeted immunohistochemistry was possible because of the use of labeled primary antibodies for tissue antigen detection. This concept laid the groundwork for all modern antibody-based spatial techniques, including both single-color and sophisticated multiplex immunofluorescence methods employed today. This became a turning point that enabled the first molecule-specific views of uneven cellular landscapes. The seminal work in this area was reported in 1941 by Coons and his colleagues at Harvard University, who conjugated anti-pneumococcus III rabbit serum with beta-anthryl isocyanate to visualize the labeled antibody under ultraviolet light [3]. While this initial work utilized unstable fluorescein isocyanate, the subsequent development of the more stable Fluorescein Isothiocyanate (FITC) in 1958 catalyzed the widespread adoption of the technique [4]. This later study marked the true inception of antibody-based immunohistochemistry (IHC), which combines anatomical, immunological, and biochemical techniques to image discrete components directly within tissues by leveraging the specific binding of labeled antibodies to their target antigens for immunohistochemical spatial detection, establishing a revolutionary principle that would evolve vertiginously over the ensuing decades. Early applications of IHC were instrumental in diagnostic pathology and basic research, enabling the detection of single-type specific proteins such as vimentin, cytokeratin, and proliferating cell nuclear antigen (PCNA) to support diagnoses and build understanding of cancer differentiation and metastases. Over the decades, continuous methodological and technological advancements have refined both bright-field IHC and dark-field immunofluorescence (IF) as sibling techniques. These improvements include enhanced tissue fixation and sectioning protocols, more robust and effective antigen retrieval protocols, and the development of superior polyclonal, monoclonal, and recombinant antibodies, as well as conjugation and immunostaining reagents. Significant progress has been made towards employing more sensitive detection chemistries, such as avidin–biotin complexes, HRP polymer-conjugated primary and secondary antibodies, high-resolution digital imaging and whole-slide automated scanning platforms. Traditional IHC and IF methods have some major limitations. First, they can only detect a small number of protein targets in a tissue sample at the same time, usually just one to four [5,6,7,8,9]. This makes it difficult to understand how cells interact with each other and how different tissues work, especially in tumors. To overcome this problem, scientists have often stained multiple tissue sections and compare them. However, this approach has its own set of problems, such as losing track of where things are in the tissue sample. It is like trying to put together a puzzle with missing pieces. As a result, it is almost impossible to understand how different cells work together and express different markers. This poses a major roadblock to understanding complex cellular interactions. The need to overcome these limitations has driven the development of new robotic systems that can dispense reagents and capture digital images of stained cells and tissue sections. This was a game-changer, allowing scientists to move beyond traditional IHC into the realm of spatial analysis. With these new systems, researchers can finally start to understand how cells interact with each other in a more detailed and accurate manner. The development of these systems was a critical step forward, opening new possibilities for understanding complex biological systems. By combining the dispensing of reagents and digital imaging, scientists can now track the co-expression of multiple markers at the single-cell level, which is a major breakthrough in different areas of biomedical research (Figure 1).

### 2.3. Beyond the Single Stain: The High-Dimensional Evolution of Multiplexed Immunofluorescence

Traditional methods for studying tissues, such as IF and IHC, have limitations that make it difficult to understand the complex biology of precision medicine [10]. To overcome this limitation, a new technique called multiplexed IHC (mIHC/mIF) was developed [11]. This method allows researchers to detect many more markers—over 40–50—in a single tissue section [12]. This provides a complete picture of the protein landscape in tissues, which is essential for deep tissue profiling. With mIHC/mIF, scientists can visualize the entire protein landscape, providing a clearer understanding of the processes occurring in the tissue. The architecture of high-plex imaging relies on several distinct engineering strategies. One prevalent methodology utilizes iterative biochemical cycles, exemplified by Cyclic Immunofluorescence (CyCIF). In this framework, a spatial map is built layer by layer through repeated rounds of antibody application, signal acquisition, and subsequent chemical quenching or stripping. Some platforms do not use chemical cycling; instead, they use molecular tagging. This means that they attach special identifiers, such as DNA barcodes or rare-earth metal isotopes, to the primary antibodies. This allows the detection of many different markers simultaneously using special optical or mass spectrometry-based tools [13,14,15]. For example, systems such as PhenoCycler and CODEX [16] use DNA barcodes, whereas MIBI uses rare-earth metal isotopes [17]. This approach enables the simultaneous detection of a wide range of markers. The field of tissue analysis is changing with the introduction of Lunaphore COMET^TM^. This new system uses a technique called sequential immunofluorescence (seqIF) to improve laboratory efficiency and allow for the analysis of many targets simultaneously [18]. What sets COMET^TM^ apart from other systems is that it does not rely on barcodes to work. Instead, it is a fully automated, high-throughput system that can analyze 40 or more targets using regular, non-conjugated primary antibodies. This approach has several advantages: For one, it speeds up the process of validating assays by eliminating the need to specially label antibodies. Simultaneously, it helps preserve tissue samples better and produces more consistent results. Overall, COMET^TM^ has the potential to make a significant impact on the field of tissue analysis by making it faster, more efficient, and more reliable. The system works very quickly, thanks to a process called fast fluidic exchange (FFeX). This technology uses small amounts of fluid to facilitate rapid reactions. Normally, it takes hours to obtain certain results, but with FFeX, it only takes a few minutes. This means that the entire tissue sample is stained evenly, which is important for obtaining accurate results. You can learn more about FFeX, which makes the process of staining tissue samples much faster and more consistent, at https://lunaphore.com/our-technology/ (accessed on 10 April 2026). Today, we have new ways to look at tissues that provide a much clearer picture of how they work. We can use special techniques, such as mIHC/mIF, to make detailed maps that show not only what kind of cells are present but also what they are doing in their specific environment. With this approach, we can see exactly how molecules interact with each other in 3D, which is a significant step forward in understanding how tissues function. The most powerful use of modern spatial biology is its ability to break down the complex organization of cells in the tumor microenvironment. By combining multiplex immunofluorescence with robust statistical modeling, we can determine the functional states of cells based on their exact location. In clinical trials, this means that we can observe in real time how immune cells interact with each other and their environment, offering a level of spatial detail that, when combined with single-cell RNA sequencing, provides a complete understanding of disease mechanisms and how patients respond to treatment. By analyzing the spatial organization of cells in the tumor microenvironment, we can gain a deeper understanding of how diseases develop and develop more effective treatments.

There is an interest to combine fluorescence (IF/dark-field) and chromogenic/enzymatic (bright-field) detection protocols. It appears that such a hybrid technique can leverage the unique strengths of both imaging modalities on a single tissue section. For example, utilization of HRP-DAB chromogenic chemistry allows the development of a permanent high-contrast image of the broader tissue architecture, especially of high-abundance structural markers easily detectable under a standard bright-field microscope. At the same time, immunofluorescence detection is aimed either at high-sensitive labeling of lower-abundance targets or at achieving precise co-localization within specific cellular compartments. The integration of both methods allows for maintaining the “morphological anchor” of a traditional chromogenic stain while exploiting the wider dynamic range and multiplexing capabilities of fluorophores with different excitation/emission spectra, effectively bridging the gap between classical histopathology and high-plex spatial biology.

## 3. Scaffolding the Future: The High-Dimensional Topography of Current Spatial Omics

Today, we do not solely focus on individual aspects, such as how the immune system fights off invaders; instead, we employ multiple methods to obtain a holistic view. This includes examining cell-to-cell communication through mRNA and the types of lipids utilized. By integrating all these components, we can understand the body’s systems in detail, aiding in understanding how different body parts function and connect in their natural environment.

### 3.1. Deciphering the Localized Transcriptome: High-Resolution Gene Mapping Beyond the Cell

Spatial transcriptomics (ST) represents a critical convergence of high-depth sequencing and classical histological precision [19]. Unlike previous genomic approaches, ST enables quantitative interrogation of the transcriptome without sacrificing the physical coordinates of gene expression. A primary methodology relies on spatially indexed arrays, a strategy pioneered by platforms such as 10x Genomics Visium. In this framework, tissue sections are placed onto a glass slide containing millions of microscopic capture spots, each embedded with a unique “spatial barcode.” When mRNA is released from the tissue, it hybridizes to these barcodes, effectively “stamping” each transcript with its original location prior to high-throughput sequencing. This method is invaluable for discovering new insights and generating hypotheses because it examines all messenger RNA in a cell, not just specific genes. This enables the creation of a comprehensive molecular map without preconceived limitations [20].

Imaging-based spatial transcriptomics is a different approach, using platforms like NanoString CosMx [21] and 10x Genomics Xenium [22]. These systems do not sequence genes like other methods; instead, they use a technique called direct fluorescent hybridization. This involves using special probes that attach to specific mRNA molecules in the tissue. High-resolution microscopy then captures images of these probes, appearing as tiny spots. This visual data helps researchers identify where specific genes are active, aiding in understanding disease mechanisms and potential treatments. The detailed visualization of gene activity across tissue areas is particularly beneficial for studying complex diseases involving numerous genes.

Choosing a spatial transcriptomics platform is a significant decision, contingent on study objectives. Even when opting for an imaging-based approach, important trade-offs remain. Considerations include the number of target genes, cell segmentation accuracy, and detection method sensitivity—all varying across commercial systems. Therefore, experimental design must balance gene analysis depth with spatial resolution, and throughput needs to optimize study outcomes.

The field of spatial transcriptomics has evolved significantly in recent years, with substantial advancements in detail and data collection. Initially, technology employed a “discrete spot” approach, examining gene expression in specific areas typically encompassing genetic information from small cell groups (1–30 cells). Although revolutionary, it provided an average view, not a detailed individual cell perspective.

Innovations like Visium HD are transforming spatial analytics [23]. They nearly eliminate gaps or biases in tissue sample analysis by reducing capture areas to 2 micrometers, creating near-complete gene maps akin to single-cell resolution. With this detail, researchers can discern tissue interfaces and cellular changes within tissues.

The field still grapples with a “trilemma” of experimental design: balancing spatial resolution, analytical throughput, and transcriptomic depth. Achieving perfection in one area often requires compromises in the others. Researchers must prioritize study goals, making difficult choices, such as focusing on small areas at the expense of data speed or gene variety. It is a trade-off dictated by research questions.

### 3.2. In Situ Proteomics: Deciphering Functional States Through High-Plex Protein Navigation

While transcriptomics provides the genetic blueprint, spatial proteomics (SP) is indispensable for capturing the actualized state of biological systems. As primary effectors of cellular function, proteins execute most biochemical processes; thus, their discrete abundance and topographical organization represent the functional ground truth of the tissue.

By mapping protein proximities and signaling hubs, researchers decode molecular logic governing disease progression and therapeutic responses. This spatial intelligence bridges the gap between “what a cell is” and “what a cell is actively doing” [24].

The adoption of spatial proteomics is driven by inherent transcript–protein discordance. Sole reliance on transcriptomics yields incomplete biological narratives, as mRNA abundance often fails to reflect actual protein concentration or enzymatic activity. Factors like translational regulation, protein half-life, and post-translational modifications render a cell’s “functional state” invisible to sequencing alone.

The necessity for direct protein interrogation was pivotal in the field’s recent formal validation. The scientific community’s shift toward high-dimensional maps culminated in spatial proteomics being named “Method of the Year 2024” [25]. This distinction endorses the technology’s maturity and its role in resolving persistent oncology and immunology challenges.

The current spatial proteomics ecosystem revolves around two operational philosophies: targeted interrogation and unbiased discovery. The targeted landscape evolves classical immunohistochemistry, employing diverse signal-amplification and encoding strategies to transcend “low-plex” limits.

Within this targeted framework, methodology typically follows one of these specialized paths:

(a) Sequential Detection: Systems like the Lunaphore COMET^TM^ utilize sequential immunofluorescence (seqIF^TM^). This automated architecture achieves hyperplexing through rapid staining and imaging cycles. Fast Fluidic Exchange (FFeX^TM^) provides subcellular resolution across multiple markers while maintaining throughput efficiency.
(b) Molecular Encoding: Other platforms bypass traditional fluorescence by using antibodies conjugated to DNA barcodes (e.g., PhenoCycler) or heavy-metal isotopes (e.g., MIBI). These “orthogonal” labels allow simultaneous detection of extensive marker panels without visible light constraints.

Despite progress, significant technical challenges remain. Fluorescence studies encounter signal mixing (spectral overlap) and tissue autofluorescence, complicating results [26]. Automation has expedited processes, but repetitive staining–imaging cycles still limit throughput and tissue viability. In addition, there is a need for affordable image manipulation software plugins [27,28].

Untargeted spatial proteomics, unlike probe-based systems, employs mass spectrometry to map molecules without pre-selected antibodies. This method measures each molecule’s unique mass-to-charge ratio, offering an unbiased tissue composition view.

A key method is MALDI Mass Spectrometry Imaging (MSI), adaptable to “top-down” (whole-protein analysis) or “bottom-up” (peptide analysis) approaches [29]. This dual capability aids in identifying proteoforms and post-translational modifications, crucial yet challenging with antibodies.

Alternatively, LC-MS-based spatial proteomics uses a “dissect-and-discover” strategy [30]. This involves precise tissue partitioning into voxels or Laser Microdissection (LMD) to isolate specific Regions of Interest (ROIs) [24]. These micro-samples undergo liquid chromatography–mass spectrometry for deep, sensitive proteomic profiles.

These methodologies suit clinical formalin-fixed paraffin-embedded (FFPE) samples, identifying novel biomarkers without assumptions. However, an “analytical bottleneck” persists: compared to high-speed multiplexing of targeted imaging (like seqIF^TM^), MS methods generally offer lower cellular throughput, necessitating a balance between unbiased proteome depth and tissue area coverage [25].

The relationship between targeted and untargeted spatial proteomics is a functional symbiosis. Targeted imaging platforms, like seqIF^TM^, excel in high-resolution adjudication of known protein landscapes—interrogating up to 50 markers with superior cellular throughput and subcellular precision.

In contrast, mass spectrometry-based architectures provide unbiased discovery, identifying novel proteoforms and post-translational modifications without pre-selected antibody constraints. The strategic convergence of these modalities, termed multi-scale spatial analytics, represents a potent translational research trajectory.

By combining these methods, researchers can transition from discovery (identifying new biomarkers broadly) to validation (detailed single-cell mapping).

### 3.3. Cross-Modality Intelligence: Synchronizing Multi-Omic Layers for High-Fidelity Tissue Mapping

The current trajectory of spatial biology departs from the “single-modality” silo. Rather than analyzing molecular layers in isolation, researchers increasingly synchronize disparate spatial “omics” data—integrating transcriptomic, proteomic, epigenomic, and metabolic layers into a unified analytical framework.

Examining tissue function comprehensively reveals complexity. By studying unique molecular signatures in native environments, we observe genetic plan execution. The integration of spatial biology, single-cell analysis, and multi-omic data, bolstered by artificial intelligence and machine learning, revolutionizes the field [31]. This shift promotes a new paradigm: spatial research shifts from precise individual measurements to understanding complex multi-level biological interactions. The goal is to advance from isolated data analysis to a comprehensive “big picture” of tissue organization. By integrating diverse data types, researchers gain deeper insights into intricate tissue structures and relationships, revealing adaptive changes.

The strategic value of this convergence is transformative. By synchronizing the genome, transcriptome, and proteome within native physical coordinates, spatial multi-omics uncovers regulatory logic governing biological function. Decoding this inter-layer crosstalk is essential for identifying precise molecular disease drivers and understanding cellular “neighborhoods” orchestrating complex physiological behaviors [25]. This comprehensive view is crucial for unraveling disease mechanisms and predicting therapeutic responses [32].

Analyzing gene-to-protein translation in specific cellular areas enhances understanding. By matching mRNA transcripts with the proteins they produce, scientists assess genetic instruction adherence in cellular regions. This is vital for understanding situations where a gene is “on” but protein production is low due to environmental signals, providing insights into specific cellular regions and gene-to-protein utilization.

A practical manifestation of this synergy is the Lunaphore COMET^TM^ workflow, bridging molecular layers on a single tissue section. Integrating RNAscope^TM^ HiPlex Pro with seqIF^TM^, the platform allows simultaneous RNA and protein signature interrogation. This automated, same-slide approach ensures perfect spatial registration at subcellular resolution, providing a high-fidelity map of the regulatory landscape governing cellular phenotype and function [33].

However, combining spatial multi-omics data presents challenges. Detail levels vary across platforms, data is often in proprietary formats, and analysis is inherently complex.Addressing these bottlenecks requires advanced bioinformatic frameworks for high-dimensional data integration. Beyond simple analysis, interoperable standards and unified data-handling protocols are essential.

Table 1 serves as a guide, consolidating key performance metrics, detection methods, and spatial resolutions of leading platforms. It compares strengths and weaknesses, establishing a common ground for research. This ensures reliable results, making a meaningful impact on lives.

## 4. From Discovery to Delivery: The Translational Impact of Spatial Multi-Omic Integration

The use of spatial “omics” is revolutionizing the way we study biology and medicine. It is giving us a detailed look at how molecules are spread out in different parts of the body. We can now see exactly where certain molecules are active, which is a big improvement over older methods that just looked at the whole body or single cells. This shift is particularly impactful in the pharmacological pipeline. By mapping drug–target interactions and cellular responses within their native microenvironments, spatial analytics are significantly reducing the timelines for target validation and clinical translation, ultimately steering the industry toward more precise, context-aware interventions.

### Molecular Cartography: Navigating the Functional Landscapes of Human Pathology

The way we understand diseases is changing thanks to spatial biology, which can show us the exact location of molecules in our bodies. This change is especially important for understanding diseases like breast cancer [34]. In the past, when we studied cancer, we would often look at a sample of cells from a tumor and think of it as just a mix of everything. But now, with new tools like fluorescent staining and spatial transcriptomics, we can see exactly where each type of cell is in the tumor and how they interact with each other. This is crucial for understanding how tumors grow and how they avoid being attacked by our immune system. By mapping out where all the different types of cells are, like the immune cells that try to fight the tumor and the cells that help the tumor hide from the immune system, we can get a much better picture of what is going on. For example, in the area around a tumor, there are many different types of cells, each with its own job. Some cells, like lymphocytes, are part of our immune system and try to fight the tumor. Other cells, like stromal cells, can actually help the tumor grow and hide from the immune system. By understanding exactly where these cells are and how they interact, we can start to decode the secrets of how tumors grow and how they evade our immune system.

- Pinpointing Subcellular Signatures: Identifying localized mRNA or protein gradients that are invisible to dissociated single-cell sequencing.
- Revealing Therapeutic Vulnerabilities: Detecting heightened expression of checkpoint inhibitors or metabolic enzymes that are spatially restricted to the tumor-stroma interface.
- Mapping Cellular Proximities: Understanding how the physical distance between effector cells and malignant clones dictates the success or failure of immunotherapy.
- The usefulness of looking at tissue in a detailed, spatial way is exemplified when it can find the specific areas that are causing cancer, which can be hidden when looking at the whole tissue.

Recently, researchers have used these methods to find very detailed patterns in many different types of diseases:

- Glioblastoma Architecture: Spatial mapping has pinpointed the focal overexpression of EphA3 and ephrinA5, revealing how these specific signaling axes coordinate tumor growth within the complex neural environment [35].
- Malignant Keratinocyte Diversity: In cutaneous squamous cell carcinoma, the identification of a unique tumor-specific keratinocyte (TSK) population has provided a new cellular target for interrupting skin cancer progression [36].
- The Metastatic Brain Atlas: In non-small-cell lung cancer (NSCLC) brain metastases, researchers have mapped a comprehensive transcriptional landscape to identify localized fibrotic drivers. This has highlighted PDGFRβ, CXCR4, and TGFB1 as critical architectural nodes that could serve as novel therapeutic targets [37,38].
- Resistance Programs in Pancreatic Ductal Adenocarcinoma (PDAC): These specialized molecular programs appear to safeguard malignant cells against standard therapies, offering a fresh trajectory for overcoming chemoresistance [39].

Spatial transcriptomics has fundamentally shifted the study of complex neurological diseases from a global analysis to a region-specific molecular interrogation.

- Proteinopathies and Demyelination: Spatial platforms are being utilized to map the progressive molecular changes associated with Amyotrophic Lateral Sclerosis (ALS), Alzheimer’s Disease (AD), Multiple Sclerosis (MS), and Parkinson’s Disease (PD) [40].
- Hippocampal Dysregulation in PD: Investigations have pinpointed distorted gene expression patterns specifically within the Parkinson’s-affected hippocampus, a region often overlooked in traditional dopaminergic-centric models [41].
- Targeting the Epilepsy Microenvironment: The discovery of the CCL5/CCR5 signaling axis as a focal driver of seizure-related inflammation demonstrates the power of spatial biology to identify precise therapeutic nodes for epilepsy [42,43].

By looking at how these changes in genes affect the body’s structure, we can gain a better understanding of not just what genes are changing but also where and why these changes lead to problems with the whole nervous system.

- Cardiac Topography: By mapping the gene expression signatures of the myocardium in situ, researchers can move beyond “bulk” tissue averages to identify the localized molecular drivers of viral myocarditis, myocardial infarction (MI), heart failure, and arrhythmogenic cardiomyopathy (ACM). When we study cells in a dish, we can miss this important interaction between the damaged and healthy tissues, which is crucial for understanding how the heart heals and remodels itself [44].
- Fibrotic Transition Nodes: By mapping SOX9 activity within the fibrotic niche, researchers can develop interventions aimed at mitigating the transition from healthy myocytes to non-functional scar tissue [45].
- Developmental Blueprints: Beyond disease, these technologies are being used to construct spatiotemporal atlases of cardiac morphogenesis, providing a high-fidelity template of how the heart’s complex four-chambered architecture is molecularly choreographed [46].
- Pathogen Topography (Decoding the Host-Response Architecture): The application of spatial biology to infectious disease research offers a high-resolution window into the molecular battlefield of the host–pathogen interface. This “architectural forensics” approach has been instrumental in characterizing the structural impact of diverse microbial threats. COVID-19 can really damage the lungs. Research helped to make a detailed map to show how the virus hurts alveoli. This map helps us understand how the virus changes the lung’s cells and causes scarring in certain areas. By studying this map, researchers can learn more about how the virus affects the lungs and find new ways to treat COVID-19 [47].
- Influenza Dynamics: Spatial transcriptomics has helped us understand how genes are turned on and off as the illness gets worse. This has allowed us to find the exact spots where our body’s natural defense system switches from protecting us to causing too much inflammation [48].
- Bacterial Microenvironments: In chronic infections like Leprosy, researchers can identify specific genes encoding antimicrobial proteins that are spatially restricted to the interface between the pathogen and the host’s immune defense [49].
- Systemic Pathophysiology (Resolving the Functional Units of the Kidney and Liver): By maintaining the spatial orientation of specialized functional units—such as the nephron or the hepatic lobule—these platforms allow researchers to move beyond organ-wide averages to pinpoint the exact coordinates of cellular dysfunction [50,51].
- Renal Pathologies: By resolving regional gene expression patterns within the glomeruli and tubules, researchers can observe how localized inflammation transitions into systemic fibrosis [52].
- Hepatic Disorders: By mapping transcriptomic and proteomic shifts across the hepatic lobule, these technologies reveal how conditions like metabolic dysfunction-associated steatohepatitis (MASH) disrupt the delicate structures affecting homeostasis [53].
- Bridging the Translational Gap: From Mechanistic Discovery to Clinical Development. This part is meant to bridge the gap between theory and practice in biological mapping and to demonstrate how spatial biology can be practically applied to discover new drugs [54]. Drug discovery is an area where spatial platforms are significantly impacting the process from identifying a potential lead to actual development. For example, spatial biology enables researchers to confirm that a candidate molecule targets the correct area and that its effects are specific to the disease being treated, rather than resulting from systemic noise. This can help de-risk the lead selection process and increase the likelihood of the drug’s effectiveness in clinical trials. Overall, this section explores how spatial biology can improve the drug discovery process and provides a roadmap for developing new, spatially informed therapeutics.

## 5. Summary

Spatial biology introduces a novel lens through which we can observe cellular functions, differing from older techniques that average large groups of cells. This approach allows for the precise visualization of cell locations and activities, moving from a mixed “soup” to a clear “syntax.” This innovative perspective reveals the fundamental rules of tissue function, influencing overall health.

By offering a detailed view of the tumor microenvironment or neurodegenerative niches, these platforms facilitate a more advanced approach to precision medicine:

**Target De-risking**: By identifying the exact expression of drug targets and the neighboring cells affecting their activity, niche-specific vulnerabilities that traditional methods may miss can be uncovered.

**Predictive Diagnostics**: Transitioning beyond simple protein abundance, topographical biomarkers that reveal the proximity of immune cells to malignant clones can predict clinical outcomes.

**Understanding complex diseases such as infections and chronic inflammation**: By examining the affected areas of the body, treatments can be developed that target specific regions, tailored to unique conditions. This approach provides a “map” to guide therapy design and create effective targeted treatments.

**The Scalability Threshold**: Overcoming Technical and Logistical Challenges

To fully realize the clinical potential of this field, it is necessary to move from isolated, single-lab experiments to industrialized, reproducible workflows. There are several key bottlenecks that must be addressed to transition spatial biology from the laboratory to clinical practice:

- **Volumetric and Resolution Frontiers**: Progressing from 2D slices to 3D volumetric reconstruction is crucial for capturing the connectivity of neural networks and vascularized tissues.
- **The Computational Tax**: The field needs to adopt interoperable data standards and cloud-native analytical pipelines to manage the extensive datasets generated by whole-organism atlases.
- **Reagent Integrity**: Standardizing reagent validation, particularly the rigorous calibration of antibodies in high-plex environments, forms the metrological basis for clinical assertions.

**Figure 2:**
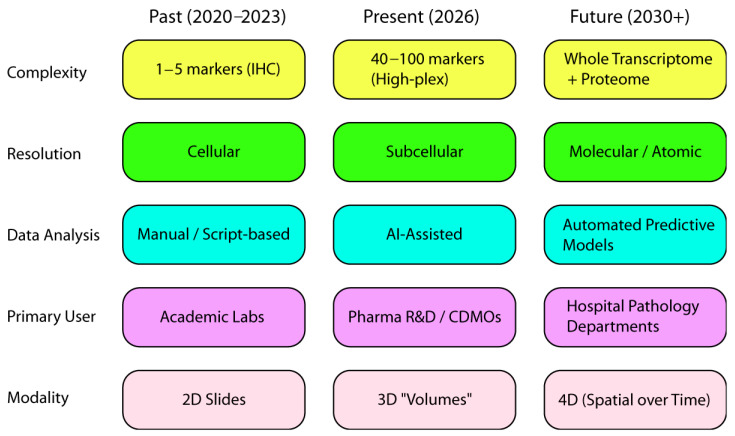
Evolution of spatial biology technology and its future trends.

The future of spatial biology (Figure 2) will be shaped by a new research paradigm that integrates various fields and industries. This approach connects academic discoveries with scalable applications in real-world settings, such as hospitals and clinics. Establishing an open, collaborative environment for sharing resources and standardizing methods is crucial. This not only facilitates the participation of new researchers but also lays the foundation for a new biology. Through collaboration and knowledge-sharing, spatial biology advances and enhances human health.

## Figures and Tables

**Table 1:** The Spatial Analytics Matrix: Distinguishing detection frameworks and research utility by technology category.

Technology Category	Platforms	Underlying Principle	Resolution	Plexity/Throughput	Key Advantages	Key Limitations
Spatial Transcriptomics (ST)	10x Genomics Visium/Xenium, NanoString CosMx, STARmap	Hybrid: Barcoded arrays (Seq) & fluorescent probes (Imaging)	Spot-based (1–50 cells) to subcellular	Hundreds to thousands of genes	Unbiased discovery; preserves complex RNA architecture.	Mostly 2D; high computational cost; subcellular “noise.”
Spatial Proteomics (Mass Spec)	MALDI-MSI, LC-MS with LMD, Deep Visual Proteomics (DVP)	Antibody-Free: Mass spectrometry profiling	Single cell to subcellular	Proteome-wide (10k+ proteins predicted)	Discovery of PTMs; no prior target info needed.	Low cell throughput; sample scarcity issues; complex prep.
Multiplex Imaging (mIF/mIHC)	CyCIF, CODEX, MIBI, Lunaphore COMET	Iterative Staining: Sequential immunofluorescence	Single cell to subcellular	Dozens (40–50+ proteins)	High cell throughput; automated; high resolution.	Antibody validation bottlenecks; tissue autofluorescence.

## Data Availability

No new data were created or analyzed in this study. Data sharing is not applicable to this article.
